# Laparoscopic Versus Open Approach for Emergency Repair of Groin Hernias: A Systematic Review and Meta‐Analysis

**DOI:** 10.1002/wjs.70076

**Published:** 2025-08-30

**Authors:** Simon D. Lai, Nicolas J. Smith, Brittany Park, Alain Vandal, Andrew D. MacCormick

**Affiliations:** ^1^ Department of Surgery Faculty of Medical and Health Sciences The University of Auckland|Waipapa Taumata Rau Auckland New Zealand; ^2^ Department of Statistics The University of Auckland|Waipapa Taumata Rau Auckland New Zealand

**Keywords:** emergency surgery, groin hernia, laparoscopic surgery, meta‐analysis, open surgery, systematic review

## Abstract

**Background:**

Although open repair has historically been the preferred approach over laparoscopic repair for acutely strangulated and incarcerated groin hernias, the laparoscopic approach is gaining popularity. This systematic review and meta‐analysis aims to investigate the safety and clinical outcomes of laparoscopic and open groin hernia repair in the emergency setting.

**Methods:**

PubMed, Embase, Scopus, Cochrane Library, and Web of Science were systematically searched for articles comparing clinical outcomes between laparoscopic and open emergency groin hernia repair in adult patients. The primary outcome was the length of hospital stay. Secondary outcomes included operative time, postoperative complications, recurrence, reoperation, postoperative mortality, and the rate of conversion from laparoscopic to open repair. Risk of bias was assessed.

**Results:**

Thirteen articles (4 prospective and 9 retrospective cohort studies) were included, with a total of 38,659 patients enrolled. Laparoscopic repair resulted in shorter length of hospital stay (MD −2.96 days [95% CI −4.91, −1.01] and *p* = 0.0074) and lower risk of wound infection (RR 0.29 [95% CI 0.20, 0.43] and *p* < 0.0001]. No statistically significant differences were observed between the two groups regarding operative time (*p* = 0.1006), risk of seroma formation (*p* = 0.3142), and risk of respiratory complication (*p* = 0.9880). Rate of conversion from laparoscopic to open repair as recorded in five studies was 2.78% ([95% CI 0.60, 11.92]).

**Conclusion:**

Emergency laparoscopic repair of groin hernias results in shorter length of hospital stay and lower risks of postoperative morbidity and mortality, with no difference in operative time when compared to open repair. Although further large‐scale prospective cohort studies and randomized controlled trials may be required to draw definitive conclusionsregarding the optimal surgical approach, laparoscopic repair of groin hernias appears to be a safe and feasible alternative to conventional open repair in the acute setting.

## Graphical abstract

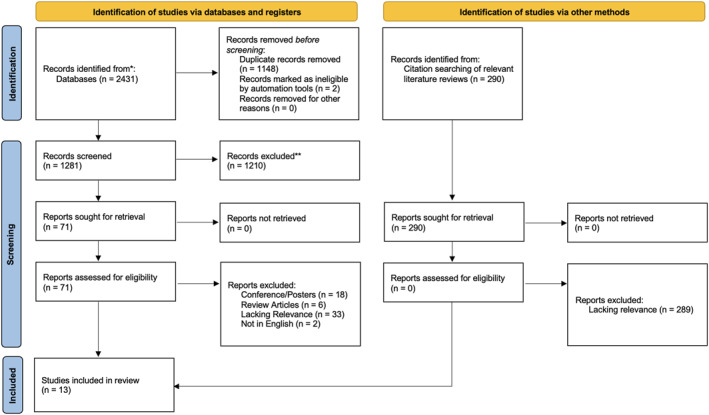

## 1 Introduction

Over 20 million patients undergo groin hernia repair globally each year, making it one of the most frequently performed surgical procedures [1]. Groin hernias can present acutely, often as strangulated or acutely irreducible hernias [2]. Acute groin hernia repair, compared to elective surgery, typically yields poorer postoperative outcomes [3]. The proportion of emergency groin hernia repairs varies significantly based on the income status of the country, with rates ranging from 3.7% to 9.6% in developed countries and reaching as high as 76.9% in developing countries [4]. Historically, open repair has been the preferred approach in emergency settings. However, in recent years, there has been a significant evolution in the landscape of acute groin hernia repair with the introduction of laparoscopic techniques [5].

Despite the increasing use of the laparoscopic technique in acute groin hernia repair, evidence regarding its efficacy compared to open repair remains limited. Consequently, the 2017 World Society of Emergency Surgery (WSES) guidelines recommends cautious consideration of laparoscopic repair, only in the absence of strangulation and the need for bowel resection [6]. The 2018 HerniaSurge guidelines advocate for a tailored approach to acute groin hernia repair based on patient condition and the expertise of the surgical team [1]. As such, there remains no definitive conclusion regarding the optimal approach to acute groin hernia repair.

This systematic review and meta‐analysis aims to investigate the differences in safety and efficacy between laparoscopic and open repair for acute groin hernias based on available evidence. Furthermore, it aims to elucidate the utility of laparoscopic surgery in acute groin hernia repair, including its potential indications and contraindications.

## 2 Material and Methods

The study was conducted according to the Preferred Reporting Items for Systematic Review and Meta‐Analyses (PRISMA) guideline [7]. The study protocol was registered on the International Prospective Register of Systematic Reviews (PROSPERO) under the registration number CRD42024486935. The PRISMA checklist is included in the supplementary material (Supporting Information S1: Appendix S1).

### 2.1 Literature Search

The PubMed, Embase, Scopus, Cochrane Library, and Web of Science electronic databases were systematically searched for studies published from database inception to 8 August 2024. The bibliography of included articles and relevant literature reviews were manually screened to identify additional eligible studies not captured by the initial search. The search strategy included a combination of terms for ‘emergency’, ‘groin hernia’, ‘laparoscopic’, ‘open’, and ‘outcomes’. Boolean operators (‘AND/OR’) were used where appropriate (Supporting Information S1: Appendix S2). No restrictions were applied on language, date, geographic location, or study design.

### 2.2 Article Selection

#### 2.2.1 Inclusion Criteria

Studies of adults aged ≥ 18 years old comparing postoperative outcomes after emergency laparoscopic and open repair of groin hernias were included. Inguinal and femoral were classed as groin hernias.

#### 2.2.2 Exclusion Criteria

Studies that include pediatric patients (< 18 years old), elective groin hernia repair or emergency repair of other types of hernia, and those reporting outcomes after emergency laparoscopic or open groin hernia repair only, without comparing the two approaches, were excluded. Letters to the editor, literature reviews, case series, case reports, and conference abstracts without full texts were excluded.

### 2.3 Study Selection

Records were exported from databases to Zotero [8] and duplicates were removed. Titles and abstracts were screened for inclusion by two authors independently using the Rayyan web application for systematic review [9]. Selected full‐texts were then derived and independently reviewed by the same two authors. Discrepancies were discussed between the two authors, with mediation with an independent senior author if required. Bibliographies for all full‐text articles included were screened as were reference lists for systematic reviews on similar subject matter.

### 2.4 Data Extraction

Data were extracted into a pro forma spreadsheet. The following categories of data were extracted: study characteristics including country, study design, year published, study period, number of participants, and follow‐up period; patient characteristics including presentation, age, sex, body mass index (BMI), American Society of Anesthesiologists (ASA) physical status classes, and laparoscopic surgical technique; and postoperative outcomes including, operative time, length of hospital stay, wound infection, seroma formation, respiratory complications, recurrence, reoperation, mortality, and conversion from laparoscopic to open surgery.

### 2.5 Risk of Bias Assessment

The Quality in Prognostic Studies (QUIPS) tool was used by two independent authors to appraise selected studies [10]. Disputes were resolved through discussion, with mediation with an independent senior author if required.

### 2.6 Outcomes Measures

The primary outcome was the length of hospital stay. Secondary outcomes included operative time, postoperative complications, recurrence, reoperation, postoperative mortality, and rate of conversion from laparoscopic to open groin hernia repair. Specific postoperative complications comprised of wound infection, hematoma formation, seroma formation, and systemic complications.

### 2.7 Statistical Analyses

All statistical analyses were performed using R version 4.3.1 (R Foundation for Statistical Computing, Vienna, Austria) [11]. Two‐tailed probability (*P*) values less than 0.05 were considered to be statistically significant.

#### 2.7.1 Meta‐Analysis

Unadjusted continuous and categorical data from univariate analyses of each study were reported as mean ± standard deviation (SD) and frequency, respectively. Continuous data reported as median and range, median and interquartile range, and mean and range were converted to mean and SD using the method of Wan et al. [12] Continuity corrections of 1 were applied to both the numerator and denominator for categorial outcomes with zero events. Pooled summary estimates were reported as relative risk (RR) for categorial outcomes and mean difference (MD) for continuous outcomes.

The random‐effects model with the DerSimonian–Laird tau estimator for between study variance was used [13]. Knapp–Hartung adjustments were used to calculate the 95% confidence interval around the pooled effects. Forest plots were used to visualize meta‐analysis results. Heterogeneity between included studies for each outcome was measured using the *I* ^2^ statistic. An *I* ^2^ value of 0%–29% was considered not significantly heterogenous; 30%–49%, moderate; 50%–74%, substantial; and 75%–100%, considerable heterogeneity. Funnel plots were assessed for a potential publication bias.

#### 2.7.2 Sensitivity Analysis

A sensitivity analysis was performed excluding one study that included 10 obturator hernias out of 106 cases to evaluate the impact of study heterogeneity [14]. The analysis was repeated using the same statistical approach.

## 3 Results

### 3.1 Search Results

**FIGURE 1:**
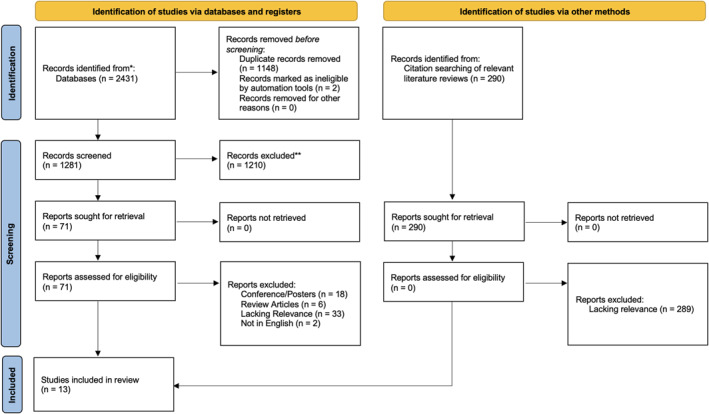
Preferred Reporting Items for Systematic Reviews and Meta‐analysis (PRISMA) flow diagram of study selection.

The database search identified 2431 records, 12 of which were included in analysis. One additional record was identified via citation searching of included studies and relevant literature reviews (Figure 1). In total, 13 articles were included in the quantitative meta‐analysis (Supporting Information S1: Appendix S3) [14, 15, 16, 17, 18, 19, 20, 21, 22, 23, 24, 25, 26].

### 3.2 Study Characteristics

The study design and characteristics are shown in Supporting Information S1: Table S1. All included studies were observational cohort studies conducted between 2001 and 2024. Three studies were conducted using data obtained from national registries and the number of centers could not be ascertained [16, 22, 23]. The remaining studies were conducted at one center.

### 3.3 Risk of Bias Assessment

The results of risk of bias assessment using the QUIPS tool are shown in Supporting Information S1: Table S2. All studies demonstrate low risk for study participation, study attrition, and statistical analysis and reporting. Four studies showed a high risk of bias for prognostic factor measurement as they did not adequately define the prognostic factors [16, 22, 23, 26]. Nine studies received moderate risk for outcome measurement as it was unclear whether outcomes were measured using validated definitions [14, 17, 18, 19, 20, 21, 22, 23, 25]. All studies demonstrated high or moderate bias for study confounding because they did not account for confounders identified.

### 3.4 Patient and Procedure Characteristics

Emergency groin hernia repairs were performed in a total of 38,659 patients. 34,983 of which underwent open surgery, whereas 3676 patients underwent laparoscopic surgery. Among the 10 studies that reported the surgical technique, 310 patients underwent transabdominal preperitoneal (TAPP) repair, whereas 55 patients underwent totally extraperitoneal (TEP) repair [14, 15, 17, 18, 19, 20, 21, 24, 25, 26]. Eight studies used the TAPP approach exclusively [15, 17, 18, 19, 20, 21, 24, 26], and two studies used a mix of TAPP and TEP repairs [14, 25]. Three studies did not report on the surgical approach as their data were sourced from an existing national registry [16, 22, 23]. Patient characteristics and preoperative variables for each study are summarized in Supporting Information S1: Table S3.

### 3.5 Outcome Measures

#### 3.5.1 Length of Hospital Stay

**FIGURE 2:**
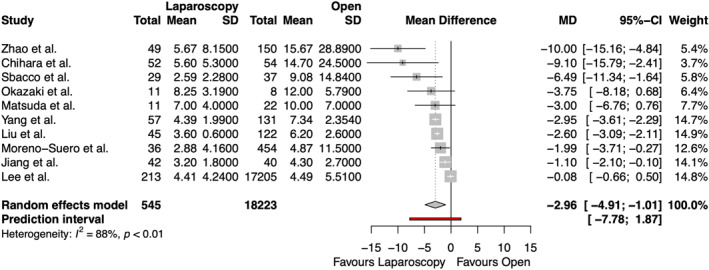
Forest plot for length of hospital stay. CI = confidence interval; MD = mean difference; SD = standard deviation.

The length of hospital stay after emergency groin hernia repair was reported in 10 studies, including 18,768 patients, shown in Figure 2. There was a significantly lower length of hospital stay after laparoscopic surgery compared to open surgery (MD −2.96 days [95% CI −4.91, −1.01] and *p* = 0.0074), although considerable heterogeneity was demonstrated (*I* ^2^ = 88.3%).

#### 3.5.2 Operative Time

**FIGURE 3:**
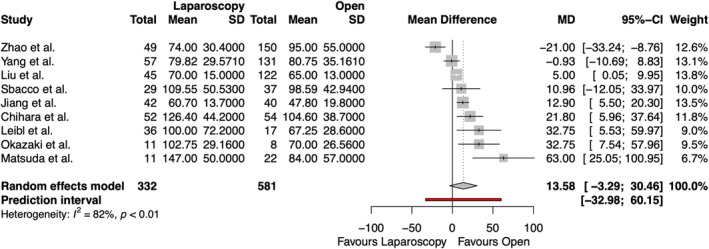
Forest plot for operative time. CI = confidence interval; MD = mean difference; SD = standard deviation.

The operative time of emergency groin hernia repair was reported in nine studies, including 913 patients, shown in Figure 3. There was no significant difference in operative time between laparoscopic and open repair (MD 13.58 min [95% CI −3.29, 30.46] and *p* = 0.1006). There was considerable heterogeneity among the studies (*I* ^2^ = 82.1%).

#### 3.5.3 Wound Infection

**FIGURE 4:**
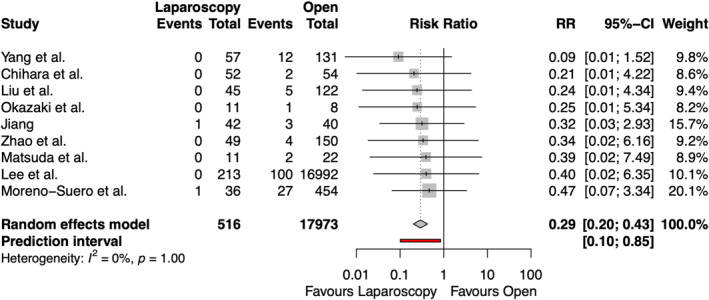
Forest plot for wound infection. CI = confidence interval; RR = risk ratio.

The rate of wound infection after emergency groin hernia repair was reported in nine studies, including 18,489 patients, shown in Figure 4. Laparoscopic repair resulted in a significantly lower rate of postoperative wound infection compared to open repair (RR 0.29 [95% CI 0.20, 0.43] and *p* < 0.0001). No heterogeneity was observed among the studies (*I* ^2^ = 0%).

#### 3.5.4 Seroma Formation

**FIGURE 5:**
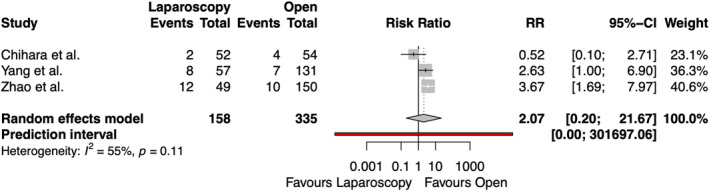
Forest plot for seroma formation. CI = confidence interval; RR = risk ratio.

The rate of seroma formation after emergency groin hernia repair was reported in three studies, including 493 patients, shown in Figure 5. No significant difference was found between the two groups regarding seroma formation (RR 2.07 [95% CI 0.20, 21.67] and *p* = 0.3142). There was substantial heterogeneity among the studies (*I* ^2^ = 55%).

#### 3.5.5 Respiratory Complications

**FIGURE 6:**
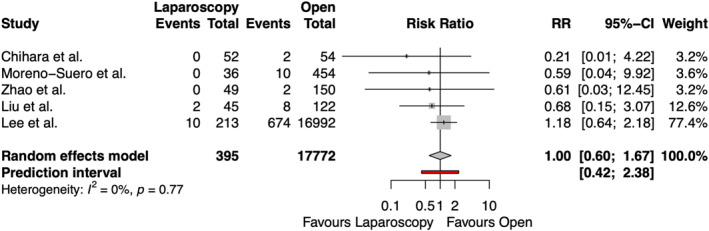
Forest plot for respiratory complications. CI = confidence interval; RR = risk ratio.

The rate of respiratory complications after emergency groin hernia repair was reported in five studies, including 18,167 patients, shown in Figure 6. One study described two and eight cases of ‘chest infections’ in the open and laparoscopic groups, respectively. This was assumed to be respiratory infections. There was no significant difference between laparoscopic and open repair in terms of postoperative respiratory complications (RR 1.00 [95% CI 0.60 and 1.67], *p* = 0.9880). No heterogeneity was observed among the studies (*I* ^2^ = 0%).

#### 3.5.6 Recurrence

Recurrence after emergency groin hernia repair was reported in four studies, including 653 patients [18, 24, 25, 26]. Due to variations in follow‐up periods across these studies, a pooled analysis for meta‐analysis was not possible. Consequently, the findings are presented in a descriptive table as shown in Supporting Information S1: Table S4. Three studies reported that there was no statistically significant difference between the laparoscopic and open groups [24, 25, 26]. One study did not report the *p*‐value [18]. However, all studies demonstrated that laparoscopic repair was associated with a lower rate of recurrence.

#### 3.5.7 Reoperation

The rate of reoperation is reported in four studies, including 10,350 patients [17, 20, 23, 24]. Different follow‐up periods were used in the studies, and a pooled analysis was not possible. The findings are presented in a descriptive table instead as shown in Supporting Information S1: Table S5. Three studies reported that there was no statistically significant difference in reoperation rates between the laparoscopic and open groups [20, 23, 24]. One study did not provide a *p*‐value [17]. However, all studies indicate that laparoscopic repair was associated with a lower rate of reoperation.

#### 3.5.8 Postoperative Mortality

Postoperative mortality was reported in eight studies, including 28,530 patients [14, 16, 17, 18, 20, 22, 23, 26]. Different follow‐up periods were used in these studies, and a pooled analysis was not feasible for meta‐analysis. The data are presented as a descriptive table instead as shown in Supporting Information S1: Table S6. Four studies concluded that there was no significant difference in postoperative mortality rate between the laparoscopic and open groups [14, 16, 20, 26]. Four studies did not report the *p*‐value [17, 18, 22, 23]. However, all studies demonstrated that laparoscopic repair was associated with a lower postoperative mortality.

#### 3.5.9 Conversion From Laparoscopic to Open Surgery

**FIGURE 7:**
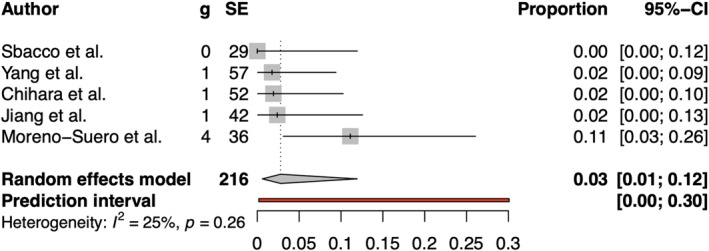
Forest plot for rate of conversion from laparoscopic and open surgery. CI = confidence interval; g = number of conversions; SE = sample size.

The rate of conversion from laparoscopic to open surgery was reported in five studies, including 216 patients who underwent laparoscopic repair, shown in Figure 7. Pooled result shows that conversion to open repair occurred in 2.78% of patients ([95% CI 0.60, 11.92]). No significant heterogeneity was observed among the studies (*I* ^2^ = 24.9%).

### 3.6 Publication Bias

Publication bias was assessed for operative time, length of hospital stay, wound infection, seroma formation, respiratory complications, recurrence, reoperation, and conversion from laparoscopic to open repair. Funnel plots are reported in Figure S1. Potential publication bias was present for length of hospital stay and operative time. Other outcomes demonstrate no apparent asymmetry on visual assessment of the funnel plots.

### 3.7 Sensitivity Analysis

One of the studies included 10 cases of obturator hernia, out of a total of 106 patients [14]. A sensitivity analysis excluding the study was performed, which did not meaningfully affect the overall results of the meta‐analysis. All previously statistically significant and nonsignificant outcomes remained unchanged. Forest plots of the sensitivity analysis are reported in Figure S2.

## 4 Discussion

The findings of this systematic review and meta‐analysis have shown that laparoscopic acute groin hernia repair results in significantly shorter hospital stays and lower risk of wound infection compared to open repair. There was no significant difference between the two approaches in terms of operative time. Based on available evidence, laparoscopic groin hernia repair appears to be a safe and feasible approach in select cases in the emergency setting.

Several included studies attributed the favorable outcome of laparoscopic repair to the utilization of diagnostic laparoscopy. Using the TAPP approach, surgeons can observe and evaluate the viability of the herniated organs. Furthermore, the time taken for hernia reduction and mesh placement allows the herniated structures to recover if possible. This allows for more thorough exploration of bowel viability and reduces the risk of unnecessary bowel resection and conversion to laparotomy, which are known to worsen patient outcomes [16, 17, 18, 19, 20, 21]. Furthermore, diagnostic laparoscopy facilitates the detection of pseudo‐incarcerations and unexpected bilateral hernias [16, 17]. One study also noted that, in patients where bowel resections are required, laparoscopic removal of adhesions before conversion minimizes the size of the incision and enhances patient outcome [16]. This supports the WSES recommendation to consider diagnostic laparoscopy to assess bowel viability if expertise is available.

Although laparoscopic repair demonstrates favorable outcomes in certain cases of acute groin hernias, results from this review do not support its widespread adoption for all presentations. Contraindications to laparoscopic repair include hemodynamic instability, significant peritoneal contamination, severe intestinal dilatation, intestinal perforation, and severe impairment of cardiopulmonary function [15, 17, 18, 19, 21, 25]. In patients where these conditions apply, there is no evidence to suggest that laparoscopic repair should be recommended over open repair. Furthermore, many studies recognize the steeper learning curve associated with laparoscopic repair, particularly in the emergency setting [15, 16, 17, 19, 20, 25]. This highlights the need for individualized assessment and management of patients presenting with acute groin hernias. The current WSES guideline recommends that strangulated groin hernias should be repaired via open approach [6]. The HerniaSurge guideline recommends a tailored approach to acute groin hernia repair, with no specification regarding indications and contraindications to laparoscopic repair [1]. Result from this review suggests that laparoscopic repair may be considered for patients without any aforementioned contraindications, where expertise is available.

This review is subject to several limitations. Foremost among these is the absence of randomized controlled trials in the current literature. All included studies are observational, resulting in low‐quality evidence due to nonrandomized study designs, biased patient allocation, and significant confounding. Notably, patients undergoing laparoscopic repair are often younger, less comorbid, and exhibit better preoperative conditions. Laparoscopic surgeries are also frequently performed by more experienced surgeons. Additionally, there is a large discrepancy between the size of the laparoscopic and open groups. Furthermore, the use and definition of clinical presentation terminologies were inconsistent. Historically, ‘incarceration’ refers to the inability to reduce the hernia mass into the abdomen, whereas ‘strangulation’ has been defined by compromised bloods supply to the herniated tissue [1]. However, these terms have been defined inconsistently across the literature. In light of this, the 2023 HerniaSurge guidelines propose updated terminology, recommending ‘acutely irreducible hernia’ for hernias that become nonreducible with acute symptoms, ‘chronically irreducible hernia’ for longstanding nonreducible hernias without new symptoms, and ‘strangulated hernia’ only when vascular compromise is confirmed by imaging or intraoperative findings [2]. The lack of standardized definitions in the existing literature limits the ability to perform meaningful subgroup analysis based on clinical presentation. This highlights the need for future studies to adopt consistent definitions for acute hernia presentations. Other limitations include the inability to compare outcomes between TAPP and TEP repairs as only two studies reported mixed use of TAPP and TEP, whereas none reported exclusive TEP use. Similarly, subgroup analysis based on different types of groin hernia is not possible due to insufficient data in the included studies. Lastly, only one study used a standardized system to grade postoperative complications [24].

In conclusion, although laparoscopic repair presents a safe and potentially superior alternative for noncomplicated presentations of acute groin hernias compared to open repair, definitive evidence is lacking. This systematic review and meta‐analysis emphasizes the need for higher‐quality evidence and hopes to guide future research endeavors and clinical practice. Ultimately, the decision between laparoscopic and open repair should be made judiciously, taking into account patient characteristics, surgical expertise, and evolving evidence‐based guidelines.

## Author Contributions

**Simon D. Lai:** conceptualization, data curation, formal analysis, investigation, methodology, validation, visualization, writing – original draft. **Nicolas J. Smith:** conceptualization, data curation, investigation, validation, writing – review and editing. **Brittany Park:** conceptualization, methodology, writing – review and editing. **Alain Vandal:** validation. **Andrew D. MacCormick:** conceptualization, methodology, project administration, supervision, writing – review and editing.

## Conflicts of Interest

The authors declare no conflicts of interest.

## Supporting information


Supporting Information S1

## Data Availability

Data sharing is not applicable to this article as no new data were created or analyzed in this study.
