# Supplementary material for: Laparoscopic Versus Open Approach for Emergency Repair of Groin Hernias: A Systematic Review and Meta‐Analysis
Source: World J Surg. 2025 Aug 30;49(10):2733–41. doi: 10.1002/wjs.70076 (PMC12515023; doi:10.1002/wjs.70076)

**Supplementary Appendices**

**Appendix S1.** Preferred Reporting Items for Systematic Reviews and Meta-analysis (PRISMA) Checklist

| **Section/topic** | **#** | **Checklist item** | **Reported on page #** |
| --- | --- | --- | --- |
| **TITLE** | | |  |
| Title | 1 | Identify the report as a systematic review, meta-analysis, or both. | 1 |
| **ABSTRACT** | | |  |
| Structured summary | 2 | Provide a structured summary including, as applicable: background; objectives; data sources; study eligibility criteria, participants, and interventions; study appraisal and synthesis methods; results; limitations; conclusions and implications of key findings; systematic review registration number. | 2-3 |
| **INTRODUCTION** | | |  |
| Rationale | 3 | Describe the rationale for the review in the context of what is already known. | 4 |
| Objectives | 4 | Provide an explicit statement of questions being addressed with reference to participants, interventions, comparisons, outcomes, and study design (PICOS). | 4 |
| **METHODS** | | |  |
| Protocol and registration | 5 | Indicate if a review protocol exists, if and where it can be accessed (e.g., Web address), and, if available, provide registration information including registration number. | 5 |
| Eligibility criteria | 6 | Specify study characteristics (e.g., PICOS, length of follow-up) and report characteristics (e.g., years considered, language, publication status) used as criteria for eligibility, giving rationale. | 5-6 |
| Information sources | 7 | Describe all information sources (e.g., databases with dates of coverage, contact with study authors to identify additional studies) in the search and date last searched. | 5 |
| Search | 8 | Present full electronic search strategy for at least one database, including any limits used, such that it could be repeated. | 5, Appendix S2 |
| Study selection | 9 | State the process for selecting studies (i.e., screening, eligibility, included in systematic review, and, if applicable, included in the meta-analysis). | 6 |
| Data collection process | 10 | Describe method of data extraction from reports (e.g., piloted forms, independently, in duplicate) and any processes for obtaining and confirming data from investigators. | 6 |
| Data items | 11 | List and define all variables for which data were sought (e.g., PICOS, funding sources) and any assumptions and simplifications made. | 7 |
| Risk of bias in individual studies | 12 | Describe methods used for assessing risk of bias of individual studies (including specification of whether this was done at the study or outcome level), and how this information is to be used in any data synthesis. | 7 |
| Summary measures | 13 | State the principal summary measures (e.g., risk ratio, difference in means). | 7-8 |
| Synthesis of results | 14 | Describe the methods of handling data and combining results of studies, if done, including measures of consistency (e.g., I^2^) for each meta-analysis. | 7-8 |

Page 1 of 2

| **Section/topic** | **#** | **Checklist item** | **Reported on page #** |
| --- | --- | --- | --- |
| Risk of bias across studies | 15 | Specify any assessment of risk of bias that may affect the cumulative evidence (e.g., publication bias, selective reporting within studies). | 8 |
| Additional analyses | 16 | Describe methods of additional analyses (e.g., sensitivity or subgroup analyses, meta-regression), if done, indicating which were pre-specified. | 8 |
| **RESULTS** | | |  |
| Study selection | 17 | Give numbers of studies screened, assessed for eligibility, and included in the review, with reasons for exclusions at each stage, ideally with a flow diagram. | 8-9, Figure 1 |
| Study characteristics | 18 | For each study, present characteristics for which data were extracted (e.g., study size, PICOS, follow-up period) and provide the citations. | 9, Table S1 |
| Risk of bias within studies | 19 | Present data on risk of bias of each study and, if available, any outcome level assessment (see item 12). | 9-10, Table S2 |
| Results of individual studies | 20 | For all outcomes considered (benefits or harms), present, for each study: (a) simple summary data for each intervention group (b) effect estimates and confidence intervals, ideally with a forest plot. | 10-15 |
| Synthesis of results | 21 | Present results of each meta-analysis done, including confidence intervals and measures of consistency. | 10-15 |
| Risk of bias across studies | 22 | Present results of any assessment of risk of bias across studies (see Item 15). | 9-10, Table S2 |
| Additional analysis | 23 | Give results of additional analyses, if done (e.g., sensitivity or subgroup analyses, meta-regression [see Item 16]). | 15-16, Figure S2 |
| **DISCUSSION** | | |  |
| Summary of evidence | 24 | Summarize the main findings including the strength of evidence for each main outcome; consider their relevance to key groups (e.g., healthcare providers, users, and policy makers). | 16-17 |
| Limitations | 25 | Discuss limitations at study and outcome level (e.g., risk of bias), and at review-level (e.g., incomplete retrieval of identified research, reporting bias). | 17-18 |
| Conclusions | 26 | Provide a general interpretation of the results in the context of other evidence, and implications for future research. | 16-18 |
| **FUNDING** | | |  |
| Funding | 27 | Describe sources of funding for the systematic review and other support (e.g., supply of data); role of funders for the systematic review. | 1 |

**Appendix S2.** Search strategy

Last updated search 08/08/2024

Total after updated search = 2431

Total after discounting retracted items and de-duplication = 1281

PubMed: 451 hits

emergen* OR urgen* OR acute* OR immediat* OR unplanned OR non-elective

AND “groin hernia*” OR “inguinal hernia*” OR “femoral hernia*” OR hernia*

AND laparoscop* OR keyhole OR “minimally invasive” OR TAPP OR TEP

AND open OR conventional

AND complication* OR morbidity OR mortality OR outcome* OR infect* OR “length of stay” OR LOS OR “length of hospital*” OR “hospital stay” OR “operative time” OR bleed* OR haematoma OR hematoma OR seroma

NOT “umbilical hernia*” OR “incisional hernia*” OR “abdominal hernia*” OR “spigelian hernia*” OR “diaphragmatic hernia*” OR “epigastric hernia*” OR “ventral hernia*” OR “hiatus hernia*” OR “hiatal hernia*”

Embase: 1046 hits

emergen* OR urgen* OR acute* OR immediat* OR unplanned OR non-elective

AND “groin hernia*” OR “inguinal hernia*” OR “femoral hernia*” OR hernia*

AND laparoscop* OR keyhole OR “minimally invasive” OR TAPP OR TEP

AND open OR conventional

AND complication* OR morbidity OR mortality OR outcome* OR infect* OR “length of stay” OR LOS OR “length of hospital*” OR “hospital stay” OR “operative time” OR bleed* OR haematoma OR hematoma OR seroma

NOT “umbilical hernia*” OR “incisional hernia*” OR “abdominal hernia*” OR “spigelian hernia*” OR “diaphragmatic hernia*” OR “epigastric hernia*” OR “ventral hernia*” OR “hiatus hernia*” OR “hiatal hernia*”

Web of Science = 498 hits

emergen* OR urgen* OR acute* OR immediat* OR unplanned OR non-elective

AND “groin hernia*” OR “inguinal hernia*” OR “femoral hernia*” OR hernia*

AND laparoscop* OR keyhole OR “minimally invasive” OR TAPP OR TEP

AND open OR conventional

AND complication* OR morbidity OR mortality OR outcome* OR infect* OR “length of stay” OR LOS OR “length of hospital*” OR “hospital stay” OR “operative time” OR bleed* OR haematoma OR hematoma OR seroma

NOT “umbilical hernia*” OR “incisional hernia*” OR “abdominal hernia*” OR “spigelian hernia*” OR “diaphragmatic hernia*” OR “epigastric hernia*” OR “ventral hernia*” OR “hiatus hernia*” OR “hiatal hernia*”

Scopus = 375 hits

(emergen*) OR (urgen*) OR (acute*) OR (immediat*) OR (unplanned) OR (non-elective)

AND (groin AND hernia) OR (inguinal AND hernia*) OR (femoral AND hernia*) OR (hernia*)

AND (laparoscop*) OR (keyhole) OR (minimally AND invasive) OR (TAPP) OR (TEP)

AND (open) OR (conventional)

AND (complication*) OR (morbidity) OR (mortality) OR (outcome*) OR (infect*) OR (length AND of AND stay) OR (LOS) OR (length AND of AND hospital*) OR (hospital AND stay) OR (operative AND time) OR (bleed*) OR (haematoma) OR (hematoma) OR (seroma)

NOT (umbilical AND hernia*) OR (incisional AND hernia*) OR (abdominal AND hernia*) OR (spigelian AND hernia*) OR (diaphragmatic AND hernia*) OR (epigastric AND hernia*) OR (ventral AND hernia*) OR (hiatus AND hernia*) OR (hiatal AND hernia*)

Cochrane Library = 61 hits

(emergen*) OR (urgen*) OR (acute*) OR (immediat*) OR (unplanned) OR (non-elective)

AND (groin AND hernia) OR (inguinal AND hernia*) OR (femoral AND hernia*) OR (hernia*)

AND (laparoscop*) OR (keyhole) OR (minimally AND invasive) OR (TAPP) OR (TEP)

AND (open) OR (conventional)

AND (complication*) OR (morbidity) OR (mortality) OR (outcome*) OR (infect*) OR (length AND of AND stay) OR (LOS) OR (length AND of AND hospital*) OR (hospital AND stay) OR (operative AND time) OR (bleed*) OR (haematoma) OR (hematoma) OR (seroma)

NOT (umbilical AND hernia*) OR (incisional AND hernia*) OR (abdominal AND hernia*) OR (spigelian AND hernia*) OR (diaphragmatic AND hernia*) OR (epigastric AND hernia*) OR (ventral AND hernia*) OR (hiatus AND hernia*) OR (hiatal AND hernia*)

**Appendix S3.** List of included studies

Chihara N, Suzuki H, Sukegawa M, Nakata R, Nomura T, Yoshida H. Is the Laparoscopic Approach Feasible for Reduction and Herniorrhaphy in Cases of Acutely Incarcerated/Strangulated Groin and Obturator Hernia?: 17-Year Experience from Open to Laparoscopic Approach. J Laparoendosc Adv Surg Tech A. 2019 May;29(5):631-637. doi: 10.1089/lap.2018.0506. Epub 2018 Oct 27. PMID: 30372373.

Jiang X, Sun R, Huang W, Yao J. Prospective comparison of two surgical approaches for incarcerated and strangulated inguinal hernia: preperitoneal hernioplasty through the lower abdominal median incision and laparoscope (TAPP). Updates Surg. 2024 Jul 17. doi: 10.1007/s13304-024-01944-x. Epub ahead of print. PMID: 39014056.

Lee Y, Tessier L, Jong A, Zhao D, Samarasinghe Y, Doumouras A, Saleh F, Hong D. Differences in in-hospital outcomes and healthcare utilization for laparoscopic versus open approach for emergency inguinal hernia repair: a nationwide analysis. Hernia. 2023 Jun;27(3):601-608. doi: 10.1007/s10029-023-02742-x. Epub 2023 Jan 16. PMID: 36645563.

Leibl BJ, Schmedt CG, Kraft K, Kraft B, Bittner R. Laparoscopic transperitoneal hernia repair of incarcerated hernias: Is it feasible? Results of a prospective study. Surg Endosc. 2001 Oct;15(10):1179-83. doi: 10.1007/s004640090073. Epub 2001 Aug 16. PMID: 11727097.

Liu J, Zhai Z, Chen J. The Use of Prosthetic Mesh in the Emergency Management of Acute Incarcerated Inguinal Hernias. Surg Innov. 2019 Jun;26(3):344-349. doi: 10.1177/1553350619828900. Epub 2019 Feb 8. PMID: 30734633.

Matsuda A, Miyashita M, Matsumoto S, Sakurazawa N, Kawano Y, Kuriyama S, Sekiguchi K, Ando F, Matsutani T, Uchida E. Laparoscopic transabdominal preperitoneal repair for strangulated inguinal hernia. Asian J Endosc Surg. 2018 May;11(2):155-159. doi: 10.1111/ases.12438. Epub 2017 Oct 19. PMID: 29052338.

Moreno-Suero F, Tallon-Aguilar L, Tinoco-González J, Sánchez-Arteaga A, Suárez-Grau JM, Alvarez-Aguilera M, Morales-Conde S, Padillo-Ruiz J. Laparoscopic vs. Open Approach in Emergent Inguinal Hernia: Our Experience and Review of Literature. J Abdom Wall Surg. 2023 Jun 16;2:11242. doi: 10.3389/jaws.2023.11242. PMID: 38515586; PMCID: PMC10955576.

Okazaki R, Poudel S, Hane Y, Saito T, Muto J, Syoji Y, Hase R, Senmaru N, Hirano S. Laparoscopic approach as a safe and effective option for incarcerated femoral hernias. Asian J Endosc Surg. 2022 Apr;15(2):328-334. doi: 10.1111/ases.13010. Epub 2021 Nov 8. PMID: 34749433.

Pina-Vaz J, Glauser P, Hoffmann H, Kirchhoff P, Staerkle R, Torney M. Inguinal and femoral hernia repair in octogenarians and nonagenarians: a population-based analysis. Int J Abdom Wall Hernia Surg. 2020 Oct-Dec;3(4):128.

Sæter AH, Fonnes S, Rosenberg J, Andresen K. High complication and mortality rates after emergency groin hernia repair: a nationwide register-based cohort study. Hernia. 2022 Aug;26(4):1131-1141. doi: 10.1007/s10029-022-02597-8. Epub 2022 Mar 29. PMID: 35348925.

Sbacco V, Petrucciani N, Lauteri G, Cossa A, Portinari M, Brescia A, Garulli G. Management of groin hernias in emergency setting: differences in indications and outcomes between laparoscopic and open approach. A single-center retrospective experience. Langenbecks Arch Surg. 2024 Jan 26;409(1):48. doi: 10.1007/s00423-024-03238-7. PMID: 38277083; PMCID: PMC10817833.

Yang GP, Chan CT, Lai EC, Chan OC, Tang CN, Li MK. Laparoscopic versus open repair for strangulated groin hernias: 188 cases over 4 years. Asian J Endosc Surg. 2012 Aug;5(3):131-7. doi: 10.1111/j.1758-5910.2012.00138.x. Epub 2012 May 20. PMID: 22776668.

Zhao F, Liu M, Chen J, Jin C, Chen F, Cao J, Liu Y. Clinical effects of prosthetic mesh in the treatment of incarcerated groin hernias. Minerva Chir. 2019 Dec;74(6):458-464. doi: 10.23736/S0026-4733.18.07824-0. Epub 2018 Oct 18. PMID: 30334396.

**Table S1.** Study Characteristics

| **Author** | **Country** | **Study Design** | **Number of centres** | **Year of Publication** | **Time period for data collection** | **Follow-up, months (mean ± SD)** |
| --- | --- | --- | --- | --- | --- | --- |
|  |  |  |  |  |  |  |
| Chihara N. | Japan | Prospective trial of lap + retrospective analysis of open | 1 | 2019 | Open: December 2000 - November 2011  Lap = December 2011 - March 2017 | NR |
| Jiang X. | China | Prospective | 1 | 2024 | January 2018 - June 2022 | Total = 34.1 ± 13.1 Open = 34.8 ± 13.8 TAPP = 33.4 ± 12.6 |
| Lee Y. | United States | Retrospective | Nation-wide database | 2023 | October 1, 2015 - December 31, 2019 | NR |
| Leibl B. J. | Germany | Prospective | 1 | 2001 | NR (6-year period) | NR |
| Liu J. | China | Retrospective | 1 | 2019 | 2009 - 2014 | Overall = 37.75 ± 20.5025 |
| Matsuda A. | Japan | Retrospective | 1 | 2017 | January 2010 - August 2016 | NR |
| Moreno-Suero F. | Spain | Retrospective | 1 | 2023 | January 1, 2011 - December 31, 2021 | NR |
| Okazaki R. | Japan | Retrospective | 1 | 2022 | April 2016 - August 2021 | NR |
| Pina-Vaz J. | Switzerland | Retrospective | Nation-wide database | 2020 | 2005-2015 | NR |
| Saeter A. H. | Denmark | Prospective | Nation-wide database | 2022 | January 1, 1998 - December 31, 2020 | Overall = 7.65 ± 5.79 |
| Sbacco V. | Italy | Retrospective | 1 | 2024 | November 2019 - September 2022 | Open = 16.54 ± 11.31 TAPP = 13.33 ± 11.11 |
| Yang GPC. | Hong Kong | Retrospective | 1 | 2012 | January 2007 - January 2011 | Open = 25.095 ± 14.48 Lap = 24 ± 15.24 |
| Zhao F. | China | Retrospective | 1 | 2019 | January 2013 - December 2017 | Overall = 26.75 ± 17.624 |

**Table S2.** QUIPS Risk of Bias Assessment

| Study | Bias domain | | | | | |
| --- | --- | --- | --- | --- | --- | --- |
|  | Study participation | Study attrition | Prognostic factor measurement | Outcome measurement | Study confounding | Statistical analysis and reporting |
| Chihara N. et al. | Moderate | Low | Low | Low | Moderate | Low |
| Jiang X. et al. | Low | Low | Low | Low | Moderate | Low |
| Lee Y. et al. | Low | Low | Low | Low | Moderate | Low |
| Leibl B. J. et al. | Moderate | Low | Low | Low | High | Moderate |
| Liu J. et al. | Low | Low | Low | Low | High | Low |
| Matsuda A. et al. | Low | Low | Low | Low | High | Low |
| Moreno-Suero F. et al. | Moderate | Low | Low | Low | High | Low |
| Okazaki R. et al. | Low | Low | Low | Low | Moderate | Low |
| Pina-Vaz J. et al. | Low | Low | Low | Low | Moderate | Low |
| Saeter A. H. et al. | Low | Low | Low | Low | Moderate | Low |
| Sbacco V. et al. | Low | Low | Low | Low | Moderate | Low |
| Yang GPC. et al. | Low | Low | Low | Low | Moderate | Low |
| Zhao F. et al. | Low | Low | Low | Low | Moderate | Low |

**Table S3.** Patient characteristics

| **Author** | **Patient Population** | **Laparoscopic Surgical Approach** | **Number of Patients** | | | **Sex, n (%)** | | **Age, years (mean +/- SD)** | | **BMI, kg/m^2 (mean +/- SD)** | | **ASA Class, n (%)** | |
| --- | --- | --- | --- | --- | --- | --- | --- | --- | --- | --- | --- | --- | --- |
|  |  |  | **Total** | **Open, n (%)** | **Laparoscopic, n (%)** | **Open** | **Laparoscopic** | **Open** | **Laparoscopic** | **Open** | **Laparoscopic** | **Open** | **Laparoscopic** |
| Chihara N. | Acutely incarcerated and strangulated groin hernias | TAPP + TEP | 106 | 54 (50.9) | 52 (49.1) | Men, n (%) = 31 (57.4)  Women, n (%) = :23 (42.6) | Men, n (%) = 27 (51.9)  Female = 25 (48.1) | 71.8 ± 15.6 | 71.4 ± 15.7 | 21.5 ± 3.6 | 21.8 ± 3.5 | I = 8 (14.8) II = 34 (63) III = 11 (20.4) IV = 0 (0) V = 1 (1.8) | I = 9 (17.3) II = 40 (76.9) III = 3 (5.8) IV = 0 (0) V = 0 (0) |
| Jiang X. | Incarcerated and strangulated inguinal hernia diagnosed by irreducible painful masses in the inguinal region and abdominal CT | TAPP only | 82 | 40 (48.8) | 42 (51.2) | Men, n (%) = 38 (95)  Women, n (%) = 2 (5) | Men, n (%) = 37 (88.1)  Women, n (%) = 5 (11.9) | 51.75 ± 2.78 | 61.17 ± 16.67 | 21.81 ± 1.84 | 22.40 ± 1.37 | I = 25 II = 9 III = 6 | I = 34 II = 5 III = 3 |
| Lee Y. | Emergency inguinal hernia repair; no further definition as the data is collected from national registry (National Inpatient Sample (NIS) data from Healthcare Cost and Utilization Project (HCUP, US) | Unspecified | 17205 | 16992 (98.8) | 213 (1.2) | Men, n (%) = 11720 (69.0)  Women, n (%) = 5272 (31.0) | Men, n (%) = 136 (63.8)  Women, n (%) = 77 (36.2) | 69.93 ± 15.68 | 68.94 ± 15.70 | Number of patients in each category, n (%):  < 30 = 16305 (96.0) 30-40 = 528 (3.1) >/= 40 = 159 (0.9) | Number of patients in each category, n (%):  < 30 = 201 (94.4) 30-40 = 9 (4.2) >/= 40 = 3 (1.4) | NR | NR |
| Leibl B. J. | Acutely incarcerated inguinal hernias | TAPP only | 53 | 17 (32.1) | 36 (67.9) | Men, n (%) = 10 (58.8)  Women, n (%) = 7 (41.2) | Men, n (%) = 23 (63.9)  Women, n (%) = 13 (36.1) | NR | NR | NR | NR | NR | NR |
| Liu J. | Acutely incarcerated inguinal hernias | TAPP only | 167 | 122 (73.1) | 45 (26.9) | Men, n (%) = 111 (91.0)  Women, n (%) = 11 (9.0) | Men, n (%) = 39 (86.7)  Women, n (%) = 6 (13.3) | 61.2 ± 18.6 | 51.4 ± 12.3 | 26.1 ± 6.1 | 25.3 ± 8.1 | NR | NR |
| Matsuda A. | Acute strangulated inguinal hernias | TAPP only | 33 | 22 (66.7) | 11 (33.3) | Men, n (%) = 14 (63.6)  Women, n (%) = 8 (36.4) | Men, n (%) = 6 (54.5)  Women, n (%) = 5 (45.5) | 71.5 ± 16.6 | 76.0 ± 11.6 | NR | NR | NR | NR |
| Moreno-Suero F. | Acutely incarcerated groin hernia | TAPP only | 490 | 454 (92.7) | 36 (7.3) | Men, n (%) = 272 (59.9)  Women, n (%) = 182 (40.1) | Men, n (%) = 24 (66.7)  Women, n (%) = 12 (33.3) | 69.15 ± 15.96 | 65.2 ± 14.47 | NR | NR | NR | NR |
| Okazaki R. | Incarcerated femoral hernias that required emergency surgery | TAPP only | 19 | 8 (42.1) | 11 (57.9) | Men, n (%) = 4 (50)  Women, n (%) = 4 (50) | Men, n (%) = 2 (18.2)  Women, n (%) = 9 (81.2) | 82 ± 6.94 | 80.25 ± 11.25 | 19.925 ± 3.34 | 20.575 ± 2.58 | I = 0 (0) II = 6 (75) III = 1 (12.5) IV = 1 (12.5) | I = 0 (0) II = 5 (45.5) III = 6 (55.5) IV = 0 (0) |
| Pina-Vaz J. | Emergency groin hernia repair; no further definition as the data is collected from national registry ("Medical statistics of hospitals" of the Swiss Federal Statistical Office) | Unspecified | 10310 | 8825 (85.6) | 1485 (14.4) | NR | NR | NR | NR | NR | NR | NR | NR |
| Saeter A. H. | Emergency groin hernia repair; no further definition as the data is collected from national registry (Danish Hernia Database) | Unspecified | 9741 | Total = 8131 (83.5) Mesh = 6331 Non-mesh = 1532 Unknown-mesh = 268 | Total = 1610 (16.5) Mesh = 1610 | Mesh:  Men, n (%) = 4793 (75.7)  Women, n (%) = 1538 (24.3)  Non-mesh:  Men, n (%) = 809 (52.8)  Women, n (%) = 723 (47.2)  Unknown-mesh:  Men, n (%) = 159 (59.3)  Women, n (%) = 109 (40.7) | Men, n (%) = 929 (57.7)  Women, n (%) = 681 (42.3) | Mesh = 67.25 ± 25.106 Non-mesh = 67.75 ± 23.3653 Unknown-mesh = 69.75 ± 23.3671 | 64.25 ± 23.95 | NR | NR | NR | NR |
| Sbacco V. | Emergency presentations of irreducible groin hernias that required emergency surgery | TAPP only | 66 | 37 (56.1) | 29 (43.9) | Men, n (%) = 21 (56.8)  Women, n (%) = 16 (43.2) | Men, n (%) = 20 (69.0)  Women, n (%) = 9 (31.0) | 77.30 ± 14.46 | 69 ± 14.43 | 36.45 ± 25.43 | 31.87 ± 21.19 | I = 2 (5.40%) II = 9 (24.32%) III = 23 (62.16%) IV = 3 (8.10%) | I = 2 (6.90%) II = 13 (44.83%) III = 14 (48.28%) IV = 0 |
| Yang GPC. | Strangulated groin hernias | TAPP + TEP | 188 | 131 (69.7) | 57 (30.3) | Men, n (%) = 115 (87.8)  Women, n (%) = 16 (12.2) | Men, n (%) = 39 (68.4)  Women, n (%) = 18 (31.6) | 74.99 ± 14.338 | 67.88 ± 14.943 | NR | NR | I & II = 89 (67.9) III & IV = 42 (32.1) V = NR | I & II = 55 (96.5) III & IV = 2 (3.5) V = NR |
| Zhao F. | Acutely incarcerated groin hernias that require emergency surgery. Emergency surgery was defined as any intervention performed within 6 hours after hospital admission. | TAPP only | 199 | 150 (75.4) | 49 (24.6) | Overall:  Men, n (%) = 161 (80.9)  Women, n (%) = 38 (19.1) | | 58.3 ± 53.3 | 55.7 ± 16.3 | 24.93 ± 16.22 | 23.3 ± 3.8 | NR | NR |

**Table S4.** Recurrence descriptive table

| **Author** | **Time restriction/follow-up period** | **Laparoscopy Group** | | **Open Group** | | **p-value** |
| --- | --- | --- | --- | --- | --- | --- |
|  |  | **Number of recurrences, n (%)** | **Total number of patients** | **Number of recurrences, n (%)** | **Total number of patients** |  |
| Liu et al. | Overall (months, mean ± SD) = 37.75 ± 20.5025 | 0 (0) | 45 | 2 (1.64) | 122 | NR |
| Sbacco et al. | Laparoscopic group (months, mean ± SD) = 13.33 ± 11.11  Open group (months, mean ± SD) = 16.54 ± 11.31 | 2 (6.90) | 29 | 3 (8.11) | 37 | 0.855 |
| Yang et al. | Laparoscopic group (months, mean ± SD) = 24 ± 15.24  Open group (months, mean ± SD) = 25.095 ± 14.48 | 1 (1.75) | 57 | 3 (2.29) | 131 | 0.815 |
| Zhao et al. | Overall (months, mean ± SD) = 20 ± 15.25 | 0 (0) | 49 | 1 (0.67) | 150 | 1 |

**Table S5.** Reoperation descriptive table

| **Author** | **Time restriction/follow-up period** | **Laparoscopy Group** | | **Open Group** | | **p-value** |
| --- | --- | --- | --- | --- | --- | --- |
|  |  | **Number of reoperations, n (%)** | **Total number of patients** | **Number of reoperations, n (%)** | **Total number of patients** |  |
| Leibl et al. | NR | 0 (0) | 36 | 3 (17.65) | 17 | NR |
| Moreno-Suero et al. | NR | 1 (2.78) | 36 | 20 (4.41) | 454 | 0.641 |
| Saeter at al. | “Any time post-operatively” | 63 (3.91) | 1610 | 474 (5.83) | 8131 | 0.3 |
| Sbacco et al. | 30 days | 1 (3.45) | 29 | 4 (10.81) | 37 | 0.291 |

**Table S6.** Post-operative mortality descriptive table

| **Author** | **Time restriction/follow-up period** | **Laparoscopy Group** | | **Open Group** | | **p-value** |
| --- | --- | --- | --- | --- | --- | --- |
|  |  | **Number of deaths, n (%)** | **Total number of patients** | **Number of deaths, n (%)** | **Total number of patients** |  |
| Chihara et al. | NR | 0 (0) | 52 | 2 (10.80) | 54 | 0.162 |
| Lee et al. | In hospital mortality during hospital stay:  Laparoscopic group (days, mean ± SD) = 4.49 ± 5.51  Open group (days, mean ± SD) = 4.41 ± 4.24 | 3 (1.41) | 213 | 319 (1.88) | 16992 | 0.714 |
| Leibl et al. | NR | 0 (0) | 36 | 1 (5.88) | 17 | NR |
| Liu et al. | Overall (months, mean ± SD) = 37.75 ± 20.5025 | 0 (0) | 45 | 2 (1.64) | 122 | NR |
| Moreno-Suero et al. | NR | 0 (0) | 36 | 10 (2.20) | 454 | 0.789 |
| Pina-Vaz et al. | NR | 11 (0.74) | 1485 | 186 (2.11) | 8825 | NR |
| Sæter et al. | 30-day mortality | 36 (2.24) | 1610 | 474 (5.83) | 8131 | NR |
|  | 90-day mortality | 50 (3.11) | 1610 | 655 (8.06) | 8131 | NR |
| Zhao et al. | Overall (months, mean ± SD) = 20 ± 15.25 | 0 (0) | 49 | 4 (2.67) | 150 | 0.574 |

**Figure S1.** Funnel plots


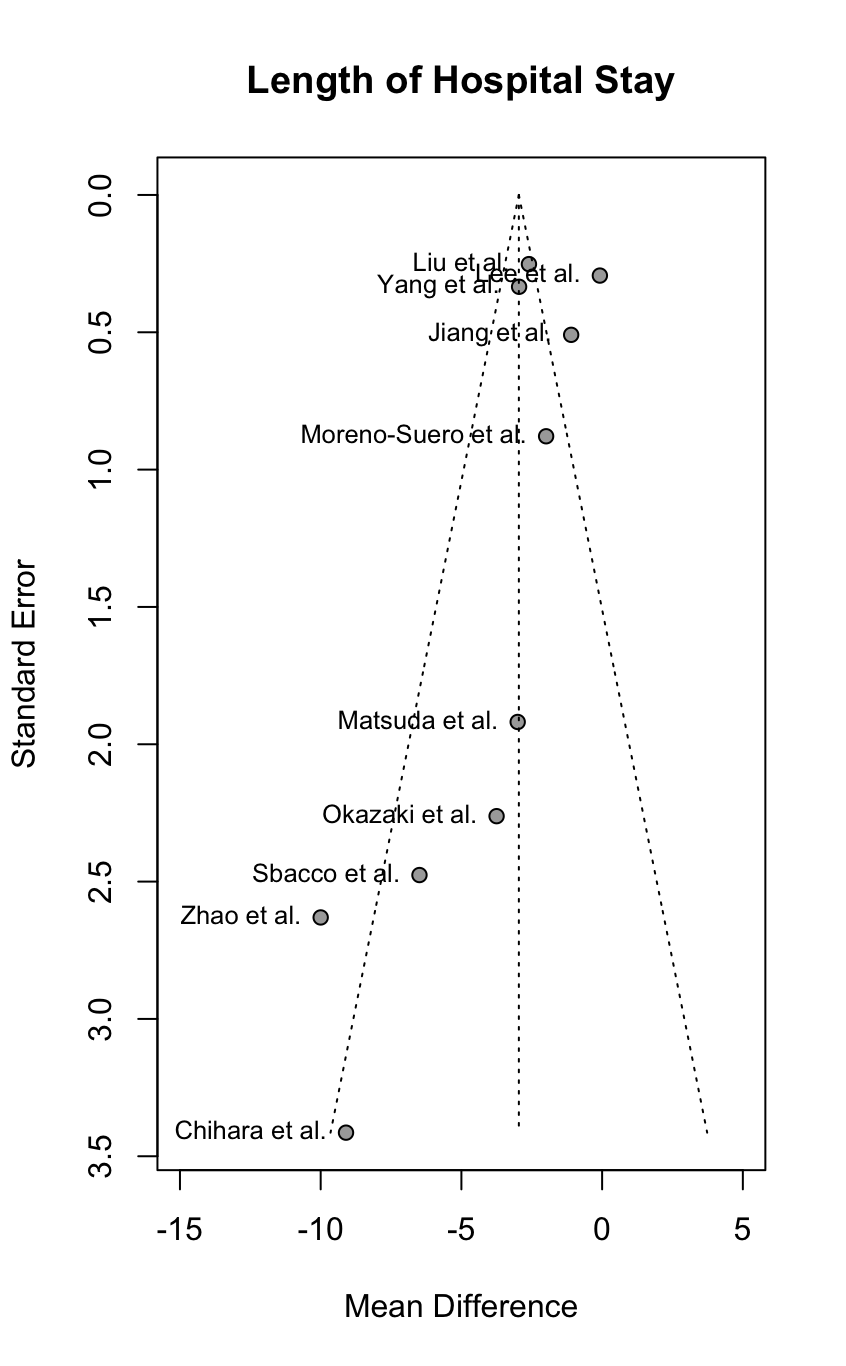

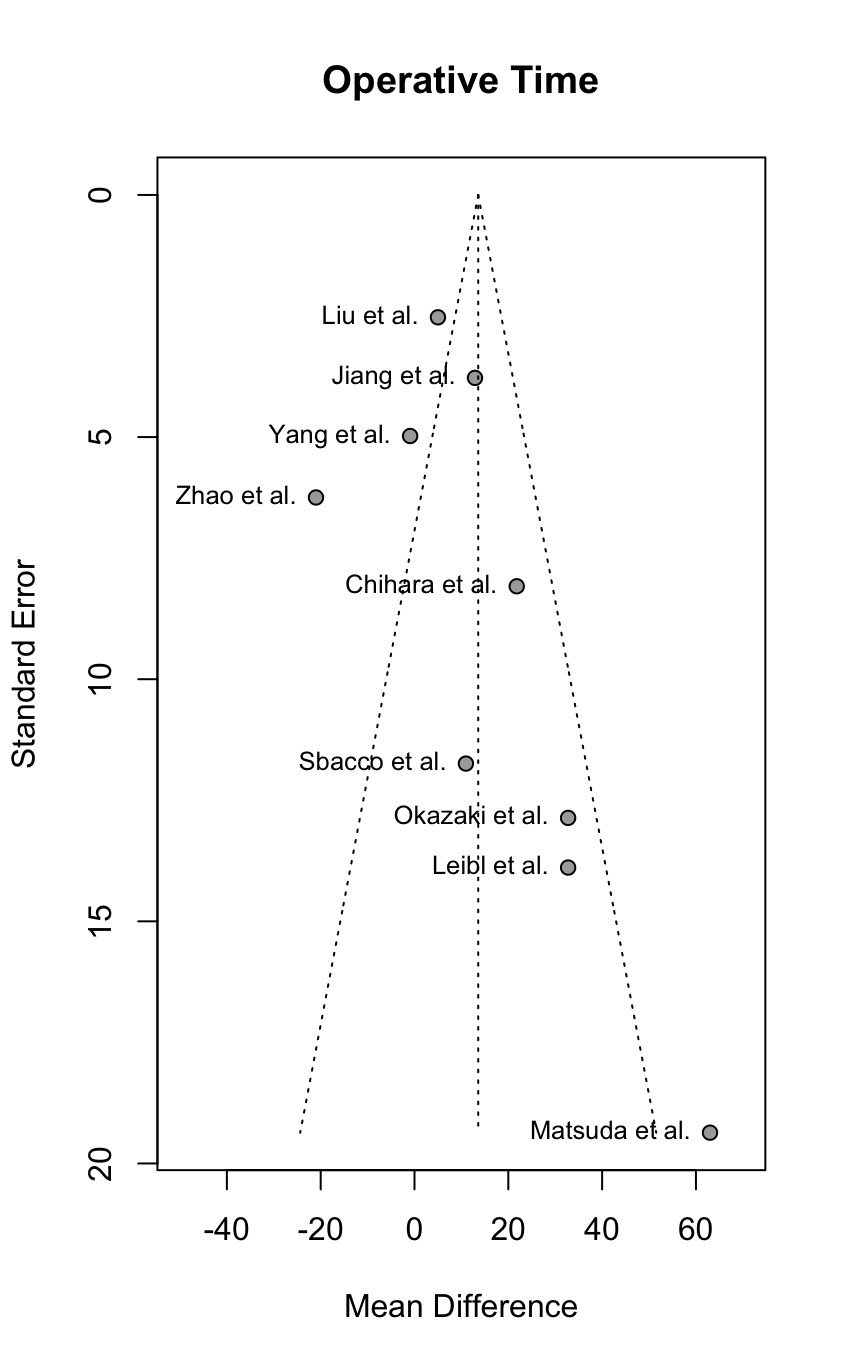

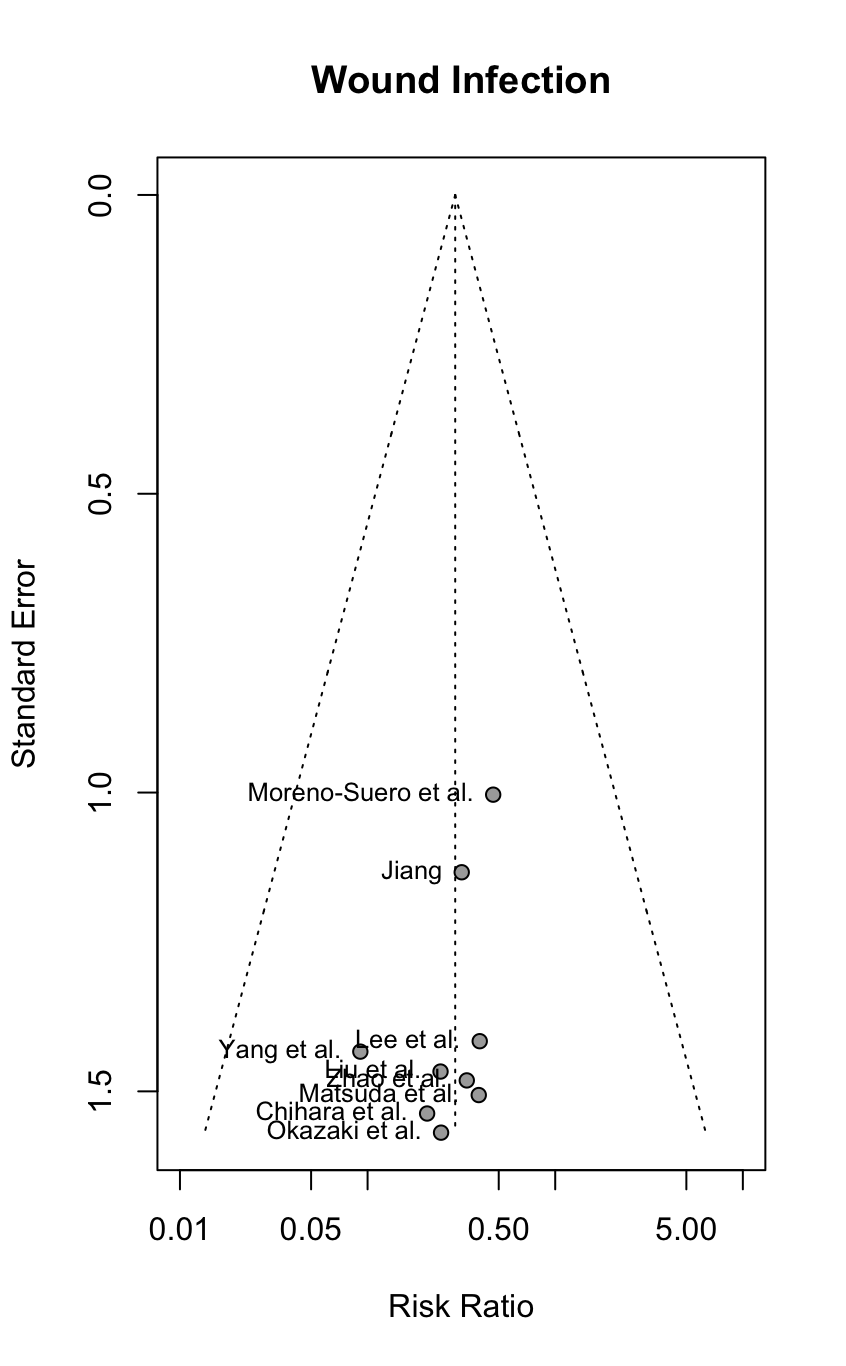


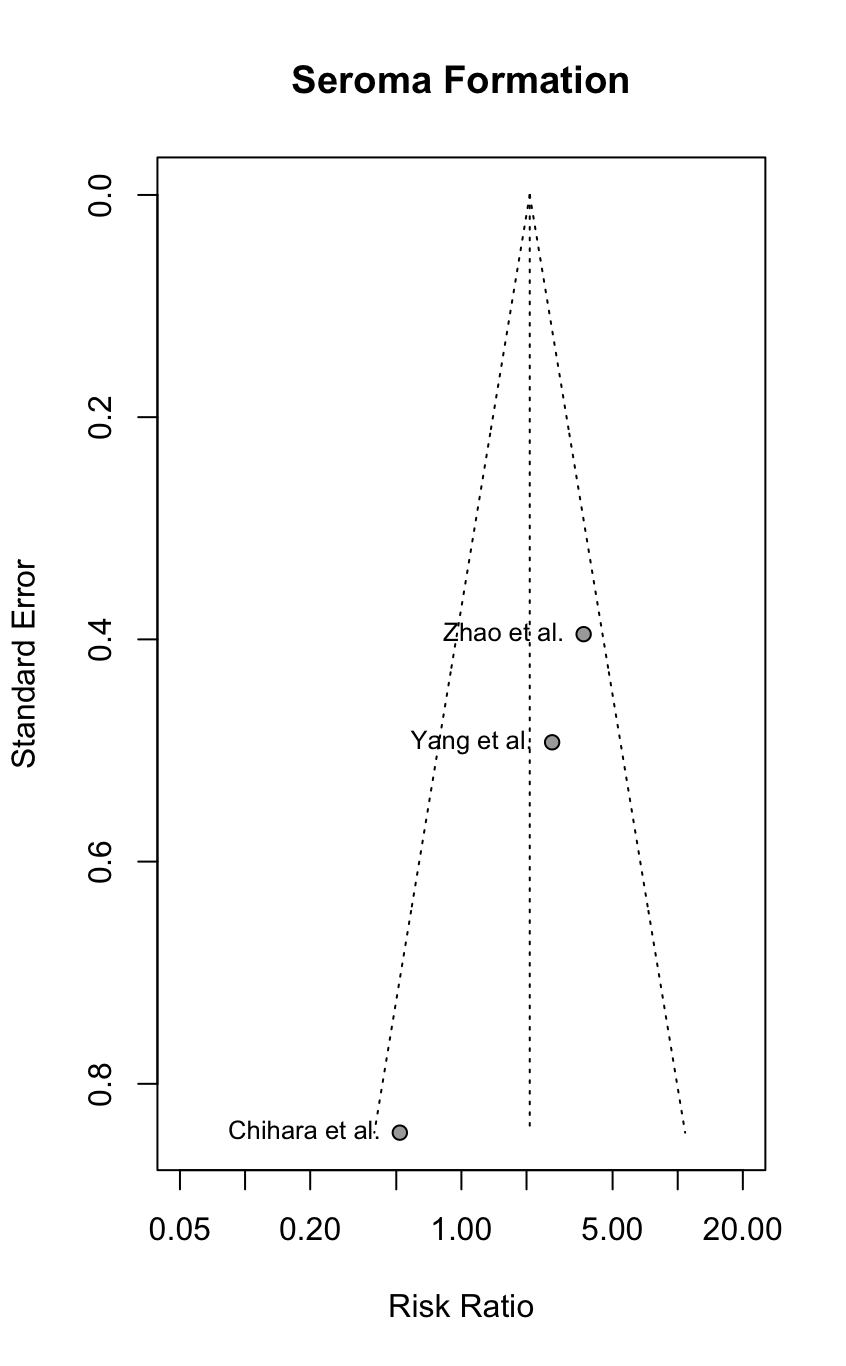

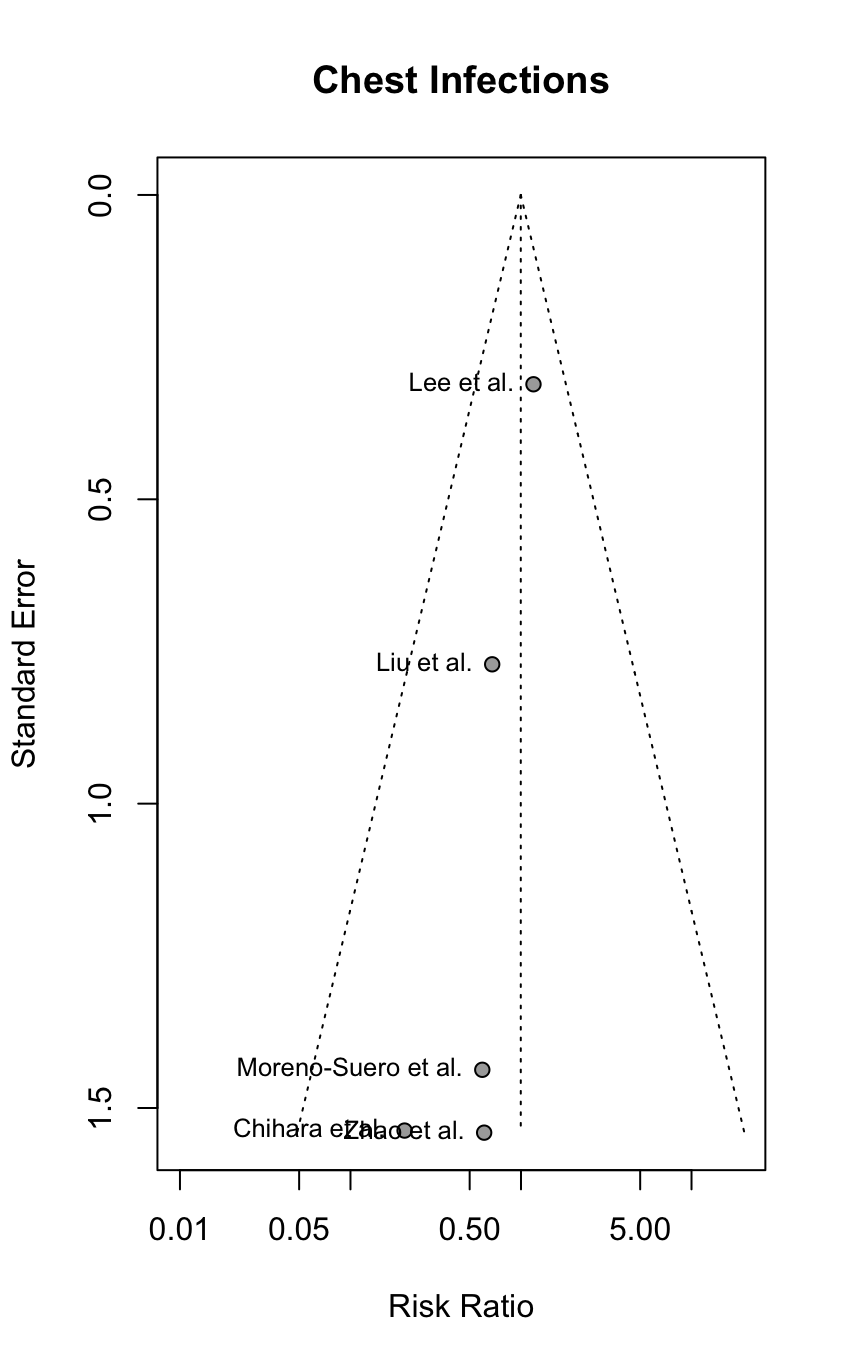

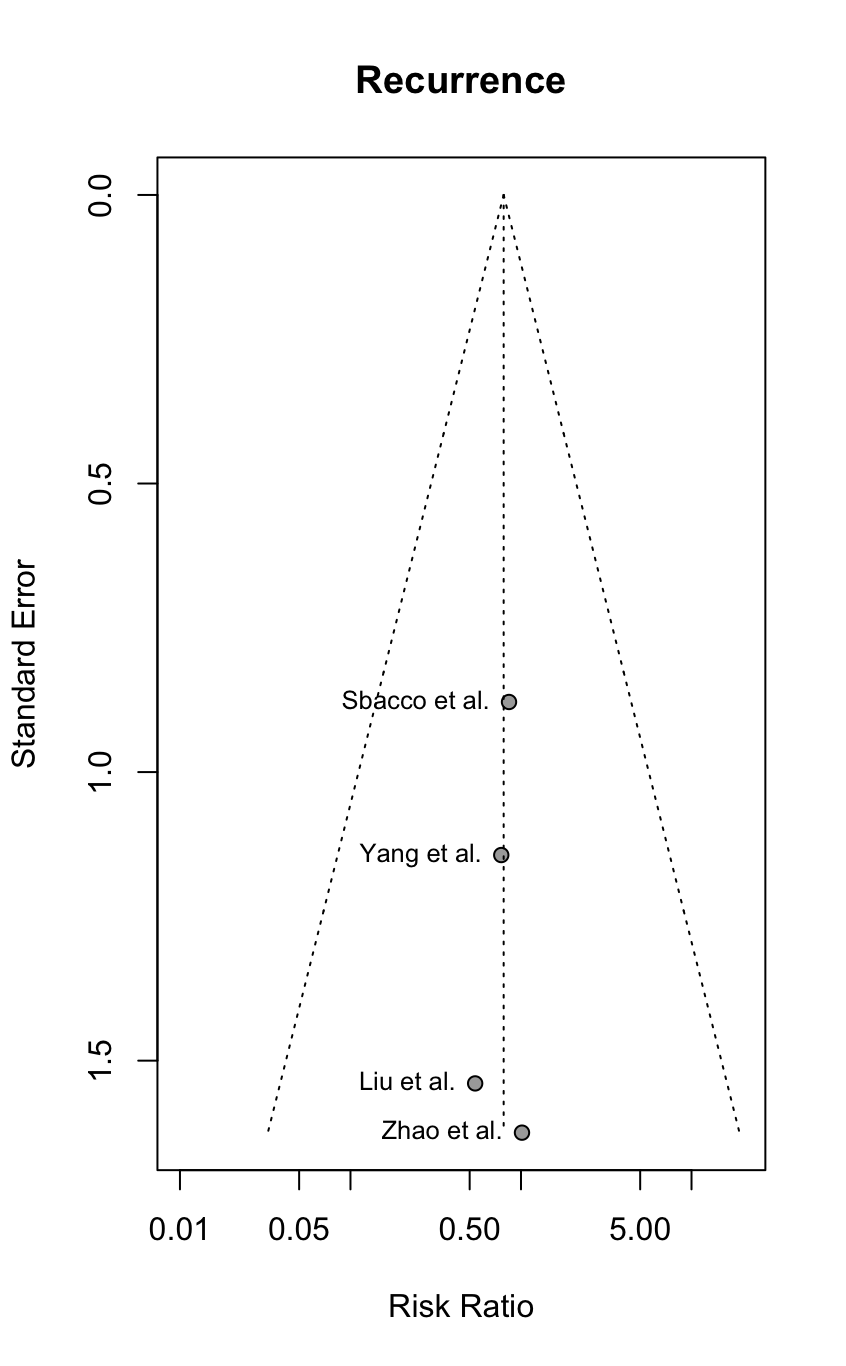

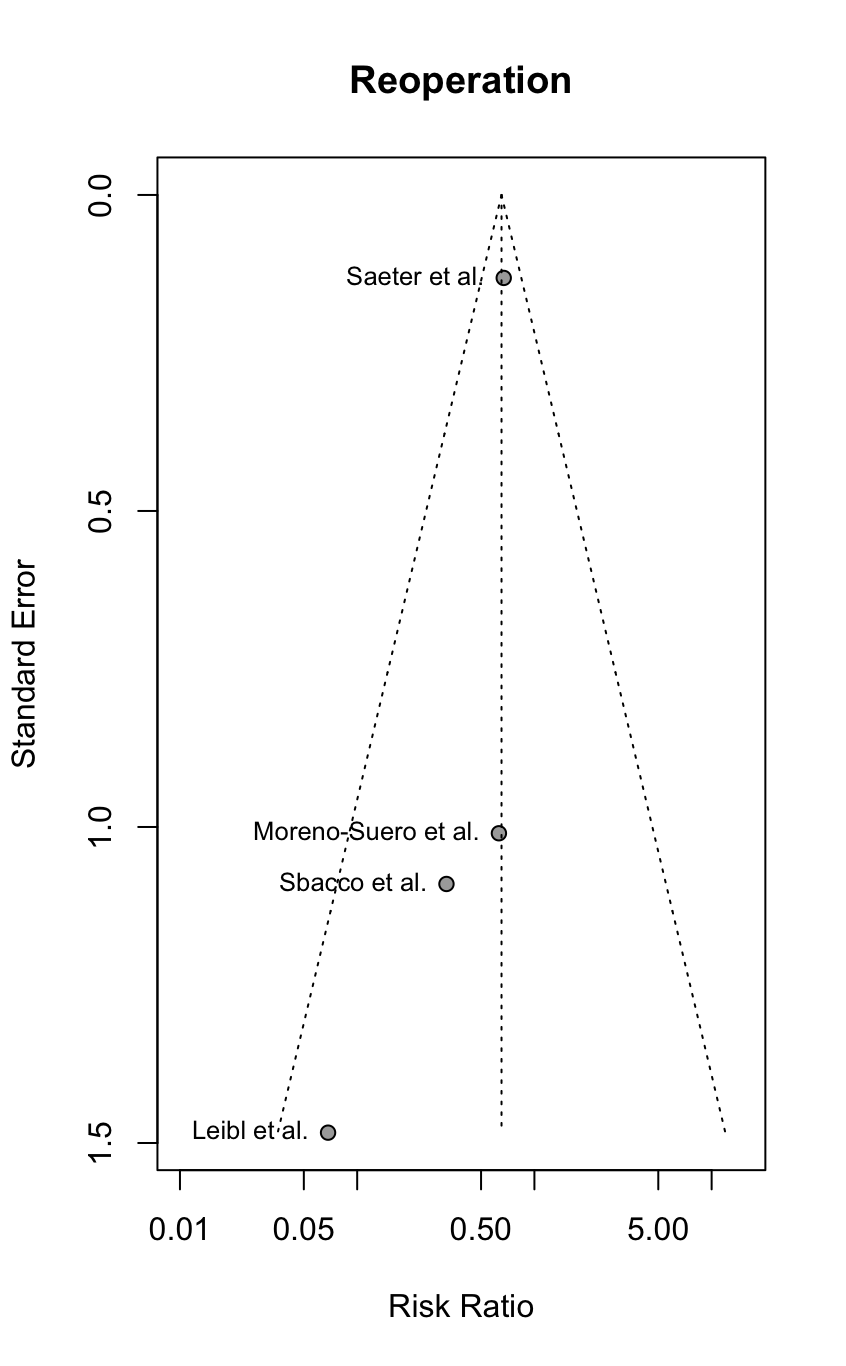

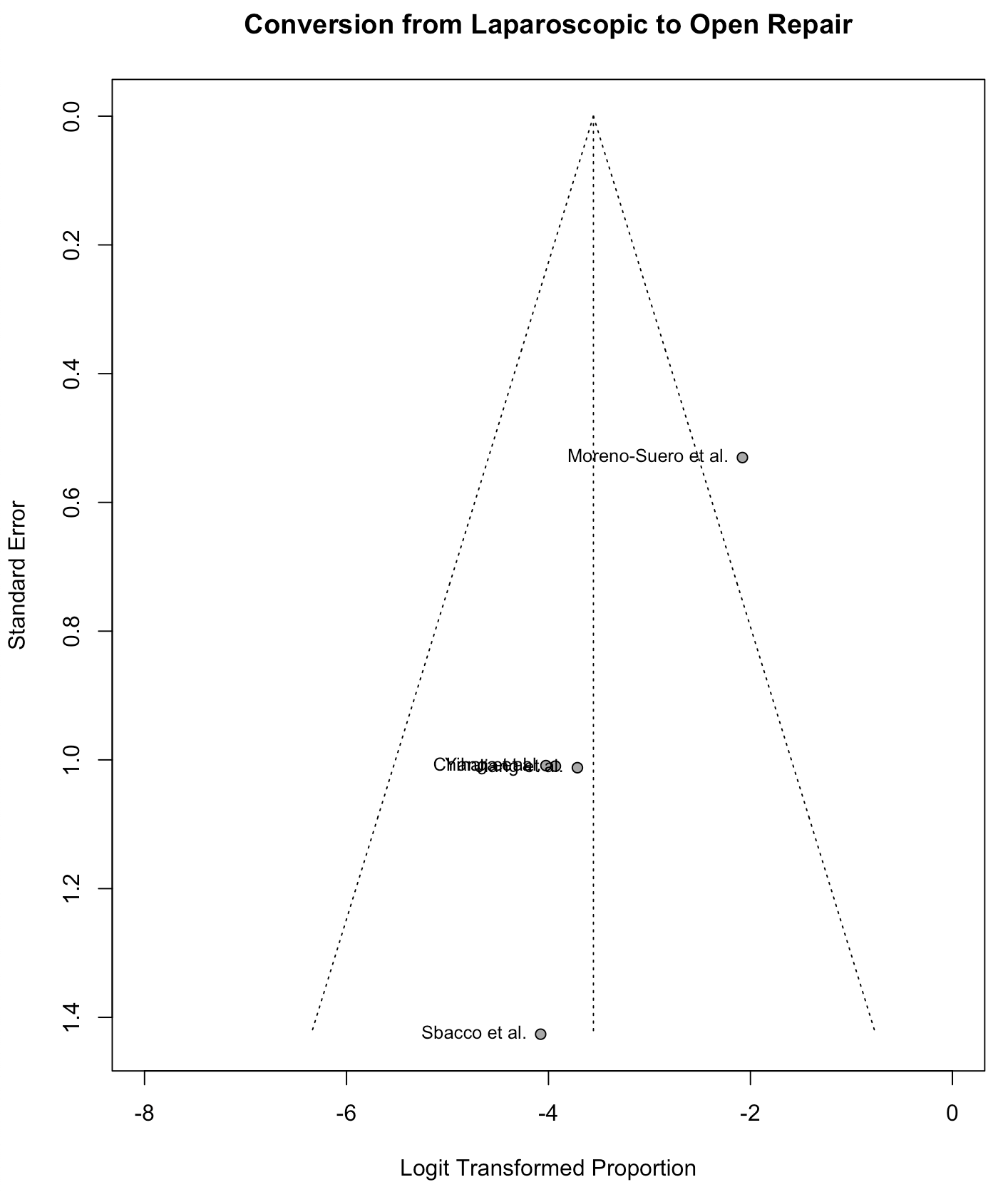


**Figure S2.** Sensitivity analysis forest plots (RR = Risk Ratio. CI = Confidence Interval)


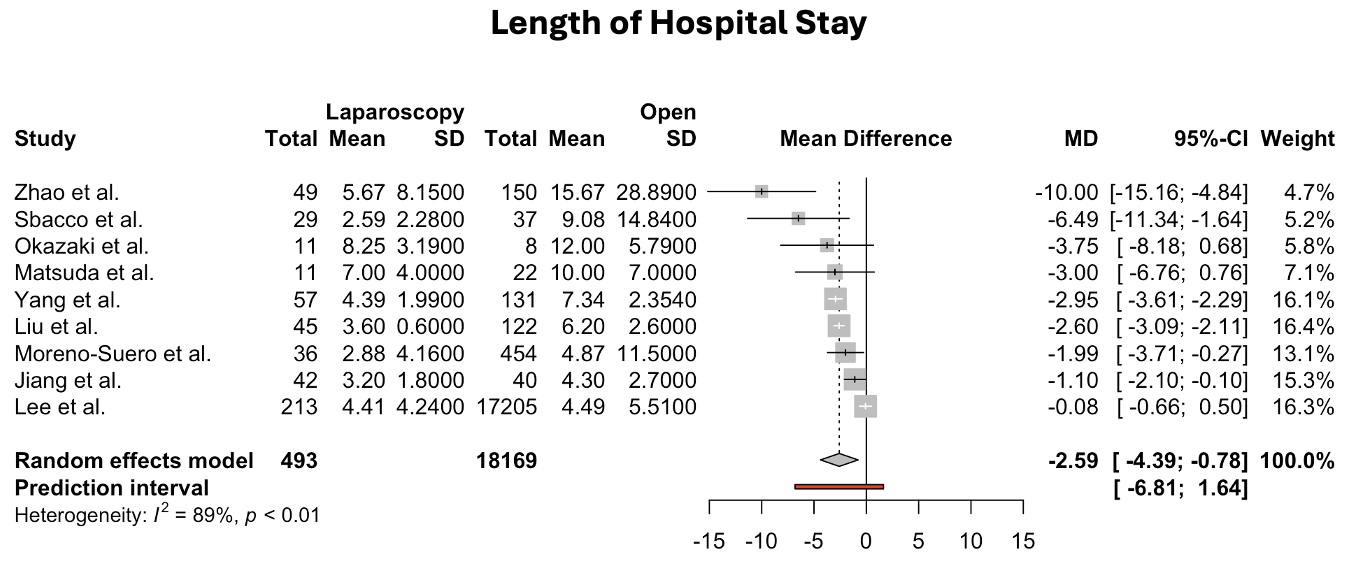


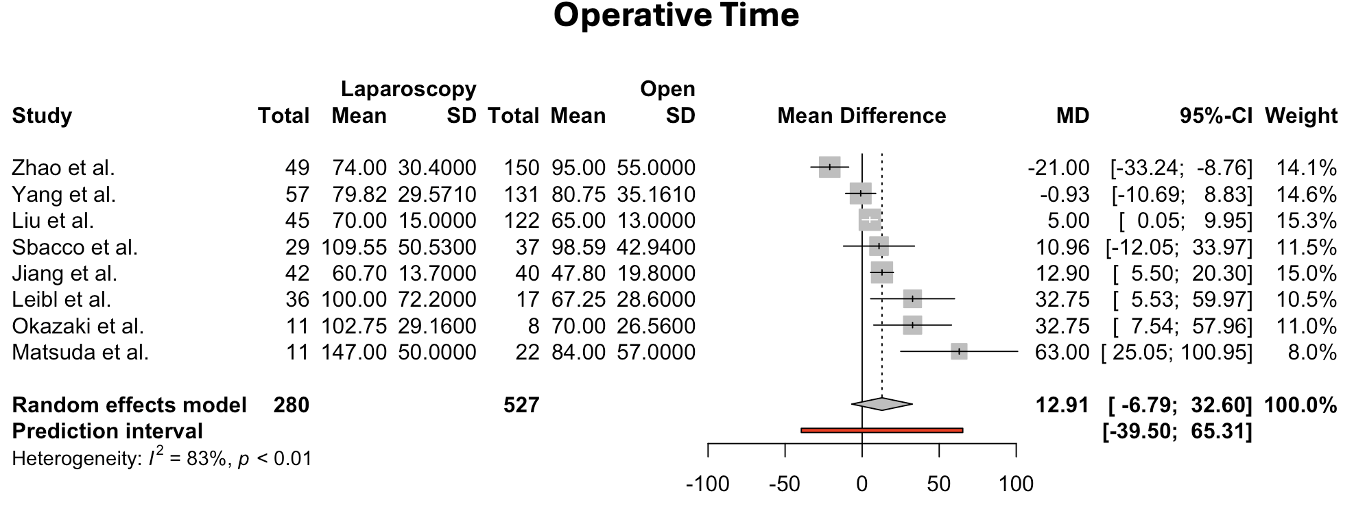


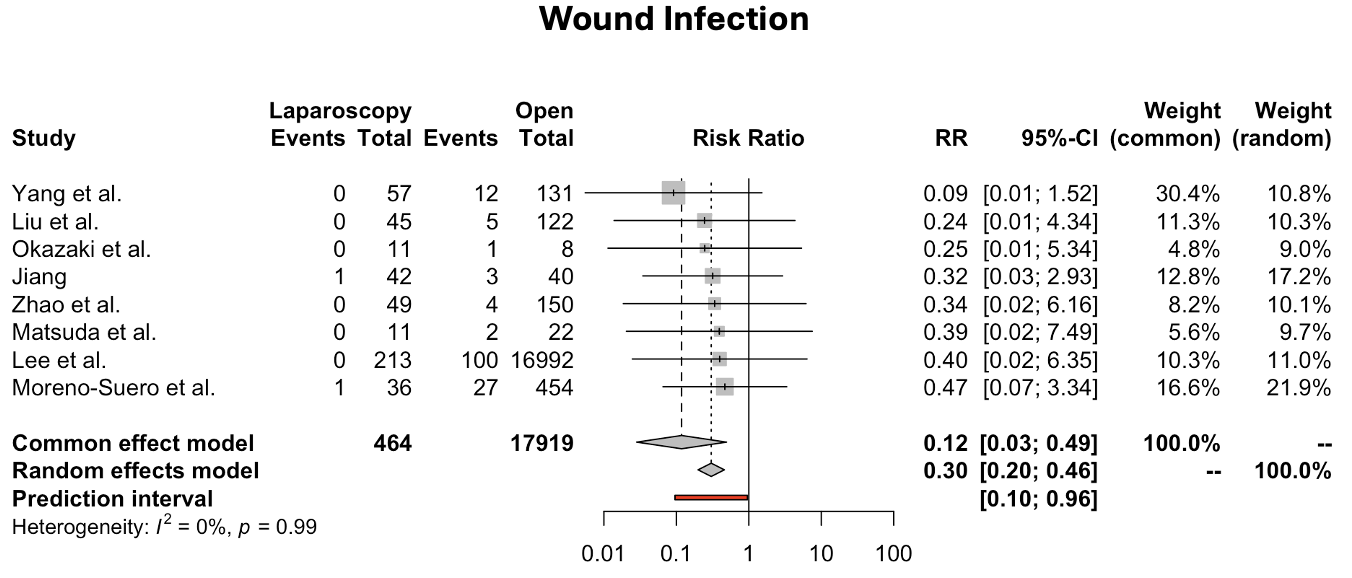


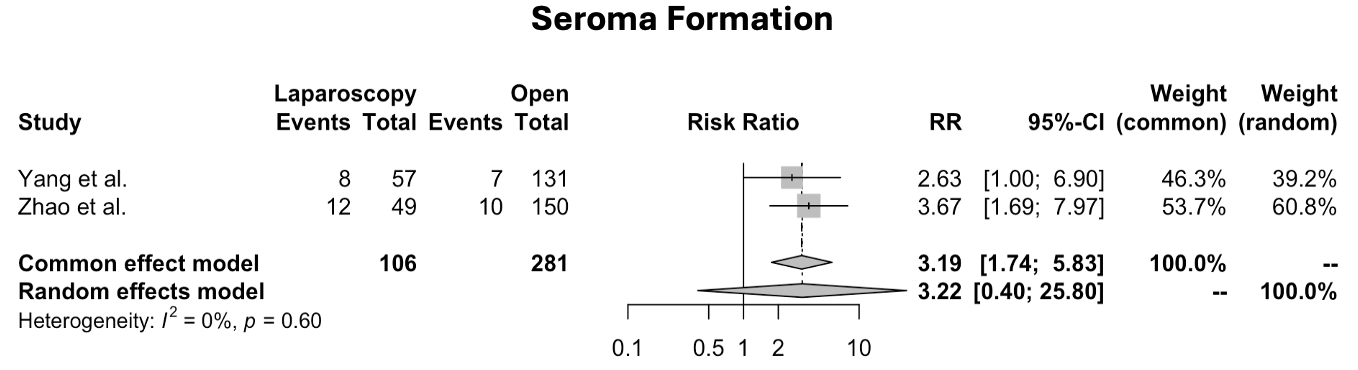


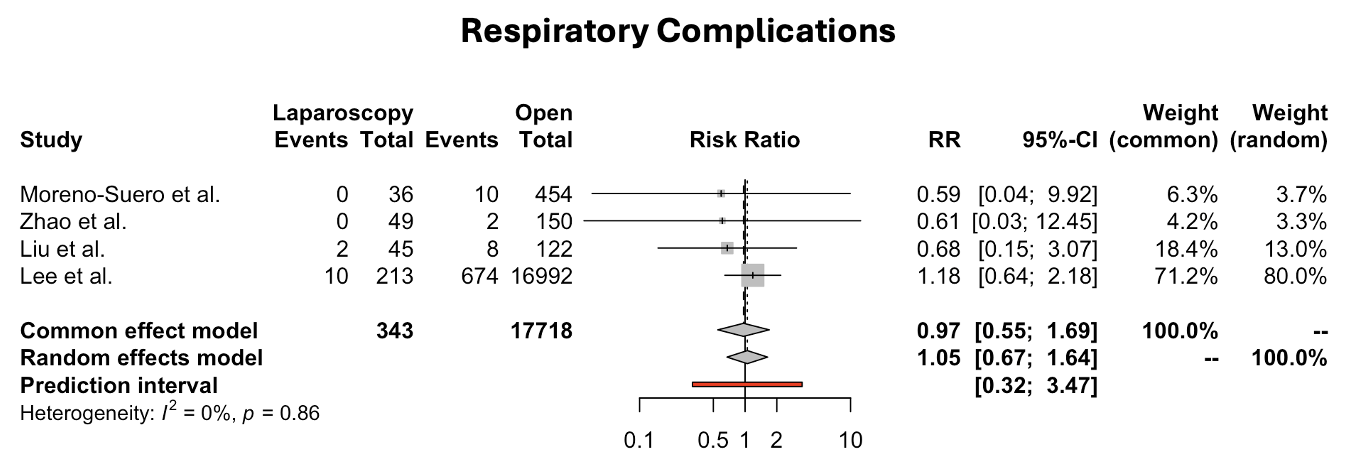


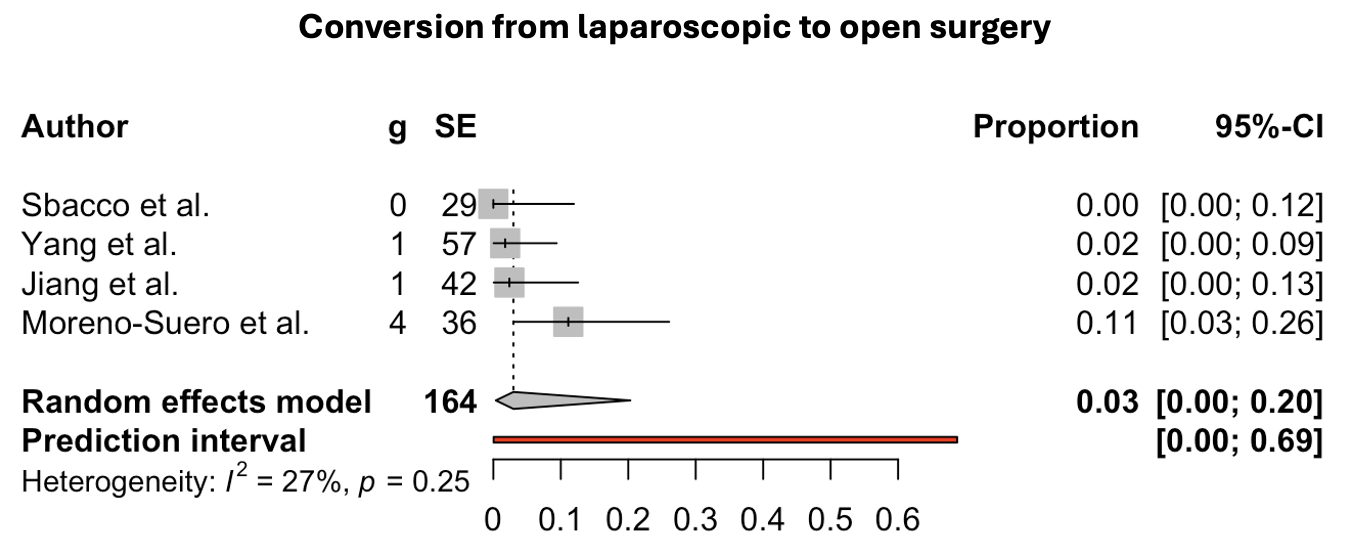

Supplement: Supplementary file 1 — Supporting Information S1 [file WJS-49-2733-s001.docx]
